# Paediatric Intussusception: A Clinical Scoring System to Predict the Risk of Operative Intervention

**DOI:** 10.34763/jmotherandchild.2020241.1934.000002

**Published:** 2020-07-29

**Authors:** Charu Tiwari, Hemanshi Shah, Gursev Sandlas, Jyoti Bothra

**Affiliations:** 1 Registrar, Department of Paediatric Surgery, TNMC & BYL Nair Hospital, Mumbai Central, Mumbai, Maharashtra, India. Pin: 400008; 2 Professor & Head, Department of Paediatric Surgery, TNMC & BYL Nair Hospital, Mumbai Central, Mumbai, Maharashtra, India. Pin: 400008; 3 Assistant Professor, Department of Paediatric Surgery, TNMC & BYL Nair Hospital, Mumbai Central, Mumbai, Maharashtra, India. Pin: 400008; 4 Registrar, Department of Paediatric Surgery, TNMC & BYL Nair Hospital, Mumbai Central, Mumbai, Maharashtra, India. Pin: 400008

**Keywords:** Intussusception, Hydrostatic reduction, Operative intervention, Score, Clinical

## Abstract

**Background:**

Intussusception is a common cause of obstruction in paediatric patients. Rapid clinical recognition and treatment is important to prevent potentially fatal complications. The present study aims to derive a clinical scoring system for prediction of risk of operative intervention in patients with intussusception.

**Materials and methods:**

Data of 100 patients with intussusception were analyzed retrospectively, and a score was calculated based on clinical parameters – age, presence/absence of symptoms and signs such as abdominal distention, vomiting, lump abdomen, red currant jelly stools and duration of abdominal pain. The maximum score was 12, and the minimum score was 6. This score was then applied to other 50 consecutive patients with intussusception.

**Results:**

Of 100, 13 patients required operative intervention; 87 patients were managed by hydrostatic reduction. In all, four patients with a score of 12 and five patients with a score of 11 required operative intervention. Seven patients had a score of 10, out of which four (57.14%) required operative intervention. A total of 87 patients who had a score of 10 or less were successfully managed non-operatively by ultrasound-guided hydrostatic reduction. In the next 50 patients, two patients with a score of 9 and all patients with scores of 10 and 11 required operative intervention. Thus, age less than 3 months and more than 2 years, presence of symptoms such as abdominal lump, red currant jelly stools and duration of abdominal pain of 2 or more days were strong predictors of operative intervention.

**Conclusion:**

This clinical score helps to predict the risk of operative intervention required in a child with a diagnosis of intussusceptions – duration of abdominal pain of 48 h or more, presence of abdominal distention and lump and red currant jelly stools are strong predictors of need of operative intervention in patients with intussusception. Higher the score (8 or more, as concluded by this study), more the probability of requiring operative intervention in these patients. Though limited, this study could serve as a pilot work to develop a user-friendly score for early surgical decision making in the management of paediatric intussusception.

## Introduction

Intussusception is the invagination of one part of the bowel into another. It is one of the common causes of acute intestinal obstruction in infants and toddlers. It is the second most common cause of acute abdominal pain in preschool children, the first being constipation (1,2). Recognition and prompt treatment of this condition is important to prevent potential complications and morbidity (1,3). Dough-nut and pseudo-kidney sign on abdominal ultrasound are diagnostic. However, the classical clinical presentation of colicky abdominal pain and vomiting with signs of red currant jelly stools and abdominal lump in a child less than 2 years of age is seen in less than 25% of children (4,5,6). This leads to delay in diagnosis (5).

Management of intussusception can be non-operative or operative. All patients after diagnosis are usually subjected to hydrostatic reduction (6). Hydrostatic reduction is done using either saline or barium; contraindications being hemodynamic instability, peritonitis, abdominal signs of perforation on abdominal X-ray and vascular compromise on USG.(ultrasonography) (6). The overall success rate of the non-operative reduction varied from 46% to 94% according to a review by Bekdash et al (6,7). As majority of patients would respond to hydrostatic reduction, it is necessary to recognise the clinical features to predict risk factors for operative treatment in these patients. Retrospective reviews in literature have presented conflicting conclusions regarding the optimal approach for managing intussusception (4).

This study aims to derive a clinical score to predict the risk of surgical intervention derived from 100 patients with intussusception, which was then applied to next 50 patients with intussusception. The study further aims to seek whether this score could be helpful in clinically predicting the patients who would be most likely to require operative intervention for intussusception at the time of admission.

## Materials and methods

This study retrospectively evaluated the data of 100 patients presenting with intussusception admitted in the Paediatric Surgery ward of a tertiary care centre from January 2011 to March 2016. The derived score was then continued to be applied on 50 more consecutive children with intussusception. After laboratory investigations, abdominal ultrasound and an erect abdominal X-ray were done to rule out complete obstruction or pneumoperitoneum. Patients with pneumoperitoneum or peritonitis were taken up for immediate exploration and were excluded from the study. Hydrostatic reduction under USG guidance was attempted for all other patients. The “rule of threes” (three attempts, each of 3 min duration and with the saline bottles at 3 ft height) was followed (3). There was a minimum time period of at least 6 h between two reductions. The patients received intravenous fluids, intravenous antibiotics, anti-spasmodics and steroids in the ward in the intervening period. The second and third attempts were tried only after confirming the viability of the bowel on ultrasound. Those patients who did not respond to three attempts of reduction or had compromised vascularity in any of the USGs underwent operative intervention.

The data of these 100 patients were retrospectively analysed, and a score was calculated for all these patients based on the following variables: age at presentation, duration of abdominal pain, vomiting, presence or absence of clinical features such as abdominal distention, abdominal lump and red currant jelly stools (Table 1). The maximum score would thus be 12, and the minimum score would be 6. The score for each of these patients was co-related with the need of operative intervention, and a *P*-value was calculated for each of these symptoms and signs.

**Table 1:** Scoring of patients with intussusception

**Score**	**1**	**2**
Age	3 months to 2 years	<3 months and >2 years
Duration of abdominal pain	<48 h	>48 h
Abdomen	Soft	Distension
Vomiting	<3 episodes	>3 episodes
Abdominal lump	Absent	Present
Red currant jelly stools	Absent	Present

This score was then applied to next 50 consecutive patients with intussusception.

## Results

A total of 100 retrospective patients were included in the study; amongst them, 13 patients required operative intervention and 87 patients were managed successfully by USG-guided hydrostatic reduction.

**Figure 1:**
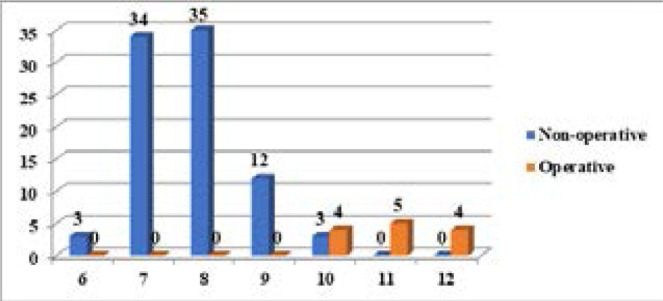
Number of patients with intussusception for each score

Four patients had a score of 12, and five patients had a score of 11; all these patients required surgery. Seven patients had a score of 10, out of which four patients required operative intervention and three were managed by hydrostatic reduction. Twelve patients had a score of 9, 35 patients had a score of 8, 34 patients had a score of 7 and three patients had a score of 6: All of them were managed by hydrostatic reduction (Figure 1 and Table 2).

**Table 2:** Scores of 100 patients and their management

**Total score**	**Total patients**	**Hydrostatic reduction**	**Surgery**	**Bowel resection for gangrene**
11–12	9	0	9	6
9–10	19	15	4	1
6–8	72	87	0	–

Seven patients had ileo-ileal or jejuno-jejunal intussusception on ultrasonography, five of whom had reduced spontaneously on observation within 48 h. Two patients with polyps on ultrasonography underwent surgery. The other 93 patients underwent USG-guided hydrostatic reduction after initial stabilisation. In all, 82 patients had successful hydrostatic reduction (four required two attempts and two required three attempts). In all, 11 warranted surgical intervention in view of failure of hydrostatic reduction with three attempts. Thus, a total of 13 patients required operative intervention.

Six underwent successful manual reduction. Two patients had palpable polyps, which were excised. One patient had Peutz–Jeghers syndrome.

Seven patients had gangrene of the bowel requiring resection – four had mesenteric lymph nodes, two had Meckel's diverticulum and one had hypertrophic payers’ patch as the lead points.

The chi-square test was applied to these 100 patients, and *P*-values were calculated for each of these variables (Table 3). *P*-values for duration of abdominal pain of 48 h or more, presence of abdominal distention, lump and red currant jelly stools were found to be statistically significant and thus strong predictors of need of operative intervention in patients with intussusception. The *P*-values for age less than 3 months or more than 2 years and presence of vomiting were not clinically significant.

**Table 3:** Analysis of *P*-value for each variable

**Score**	**1**			**2**			**P-value**

**No. of patients**	**Total**	**Hydrostatic reduction**	**Required surgery**	**Total**	**Hydrostatic reduction**	**Required surgery**	
Age	58	53	5	42	34	8	0.1259
Abdominal pain	73	71	2	27	16	11	<0.0001
Per abdomen (soft/distended)	77	75	2	23	12	11	<0.0001
Vomiting	20	20	0	80	67	13	0.0533
Lump	88	86	2	12	1	11	<0.0001
Red currant jelly stools	67	65	2	33	22	11	<0.0001

**Figure 2:**
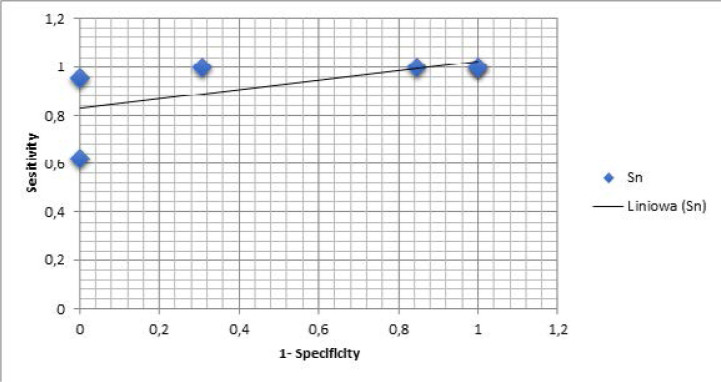
The receiver operating curve for 100 patients, which comes for score of 8

Receiver operating curve analysis was done for the score of 8 (Figure 2). The area under the curve was 0.8, suggesting that the score of 8 was found to be good at separating patients who would or who would not require operative intervention.

We continued to apply this score on 50 prospective patients with intussusception – there were nine patients with score of 6, 25 patients with a score of 7, six patients with a score of 8, six patients with a score of 9, two patients with a score of 10 and two patients with a score of 11 (Table 4).

**Table 4:** Scores of 50 prospective patients and their management

**Total score**	**Total patients**	**Hydrostatic reduction**	**Surgery**	**Bowel resection for gangrene**
10–12	4	0	4	4
9	6	4	2	2
6–8	40	40	0	–

Two patients with a score of 9 and all patients with scores of 10 and 11 – total six patients – required operative intervention. All patients required resection and anastomosis. Two patients had ileo-caecal intussusception with Payers’ patches as lead point and ileal gangrene and one had Meckels’ diverticulum as the lead point. Three patients had ileo-ileal intussusception with polyps as lead point. Histopathology suggested non-Hodgkin's lymphoma with negative resection margins. Both are currently under chemotherapy. Thus, a score more than 8 was observed to be a very strong predictor for operative intervention. All patients with a score of 8 or less were observed to have responded to non-operative management.

## Discussion

Intussusception is one of the common causes of acute intestinal obstruction in infants and toddlers and the second most common cause of acute abdominal pain in preschool children after constipation (2). Not all children with intussusception require operative intervention; majority would get reduced by non-operative methods. So, it is important to clinically identify the risk factors predicting surgery so that the intussuscepted bowel can be salvaged.

Most of the cases of intussusception (75%) occur in the first 2 years of life (3). Perinatal intussusception in newborns is more likely to be caused due to a pathologic lead point (3). Such patients require surgery for removal of this lead point. Mere hydrostatic reduction would not be able to cure pathological lead points. Similar is the case with children older than 2 years who have a higher risk of having a pathologic lead point. This may lead to recurrences after hydrostatic reduction warranting operative intervention. The incidence of intussusception caused by a PLP (Pathological Lead Point) is known to increase with age from about 5% in the first year to 44% within the first 5 years of life and 60% in 5- to 14-year olds (3).

Children with age between 3 months and 2 years are more likely to have idiopathic intussusceptions, which has high probability of being successfully managed by non-operative intervention like hydrostatic reduction. Intussusception should ideally be suspected when a child presents with any of the two classic symptoms (abdominal pain or vomiting) or two classic signs (abdominal mass or rectal bleeding) (3). Though most of the children present within 24 h of the onset of symptoms (3), delayed presentation is not rare in a developing country like ours. Abdominal lump and rectal bleeding are signs of delayed presentation, and such probability of non-operative management to be successful in these children decreases. The last sign to occur is per rectal bleeding, and prolapse of intussusceptum per rectally is a grave sign (3). Thus, abdominal lump and rectal bleeding at admission are risk factors requiring surgical intervention.

Several factors such as younger age, rectal bleeding, radiological signs of intestinal obstruction or longer duration of signs and symptoms (>72 h) have been implicated in the failure of hydrostatic reduction (3). However, presence of these signs and symptoms does not preclude hydrostatic reduction, provided the patient is well hydrated and clinically stable (3).

Other studies have found variable results while analysing the potential predictors of the need for operative reduction in paediatric intussusception patients (4,8,9,10,11,12,13). Some studies have reported that rectal prolapse, a longer duration of symptoms, bloody diarrhoea and dehydration are predictive of the need for surgery, while conversely one study found that the length of symptoms was not predictive of surgery (4,8,9,10,11,12,13). However, in this study, it was realised in retrospect that all these factors were present in our patients and have a role in prognosis and outcome of these patients. So, the present score was derived based on all these factors. The present score tries to predict the probability to require operative intervention. The higher the score, the greater the fact that the intussusception in these patients is pathological and/or has been delayed beyond non-operative reduction so that operative reduction is necessary.

However, limitations of this study cannot be ignored in view of less number of patients and methodology limitations. It may serve as a pilot work to develop a score in a larger population-based study.

## Conclusion

This clinical score helps to predict the risk of operative intervention required in a child with a diagnosis of intussusceptions – duration of abdominal pain of 48 h or more, presence of abdominal distention, lump and red currant jelly stools are strong predictors of need of operative intervention in patients with intussusception.

Higher the score (8 or more, as concluded by this study), more the probability of requiring operative intervention in these patients. Though limited, this study could serve as a pilot work to develop a user-friendly score for early surgical decision making in the management of paediatric intussusception.
